# Recent Progress in the Methodologies to Identify Physiological Ligands of Siglecs

**DOI:** 10.3389/fimmu.2021.813082

**Published:** 2021-12-10

**Authors:** Huei-Syuan Jiang, Shao-Chien Zhuang, Chak Hin Lam, Lan-Yi Chang, Takashi Angata

**Affiliations:** ^1^ Institute of Biological Chemistry, Academia Sinica, Taipei, Taiwan; ^2^ Chemical Biology and Molecular Biophysics Program, Taiwan International Graduate Program, Academia Sinica, Taipei, Taiwan; ^3^ Institute of Biochemical Sciences, National Taiwan University, Taipei, Taiwan; ^4^ Genomics Research Center, Academia Sinica, Taipei, Taiwan

**Keywords:** Siglec, ligand, glycotope, counter-receptor, proximity labeling, cell array, genome-wide knockout/knockdown screening

## Abstract

Siglecs, a family of receptor-like lectins, recognize glycoproteins and/or glycolipids containing sialic acid in the extracellular space and transduce intracellular signaling. Recently, researchers uncovered significant contributions of Siglecs in cancer immunity, renewing interest in this family of proteins. Previous extensive studies have defined how Siglecs recognize glycan epitopes (glycotopes). Nevertheless, the biological role of these glycotopes has not been fully evaluated. Recent studies using live cells have begun unraveling the constituents of Siglec ligands. These studies demonstrated that glycoprotein scaffolds (counter-receptors) displaying glycotopes are sometimes just as important as the glycotope itself. These new insights may guide future efforts to develop therapeutic agents to target the Siglec – ligand axis.

## Introduction

Siglecs (sialic acid-binding immunoglobulin superfamily lectins) are a family of type-I transmembrane proteins belonging to the immunoglobulin superfamily (1–5). Most of them are expressed on one or more subsets of leukocytes, and participate in signal transduction by regulating the tyrosine phosphorylation/de-phosphorylation cycle of signal transduction molecules. This regulation is achieved by recruiting tyrosine phosphatase SHP-1 or tyrosine kinase Syk in the cytoplasm. Recent studies have shown that some Siglecs expressed on killer leukocytes (such as Siglec-7 on natural killer cells and Siglec-9 on cytotoxic T cells of tumor patients) work similarly to classical immune checkpoint receptors (e.g., programmed cell death protein-1) (6, 7), and some others expressed on phagocytes (e.g., Siglec-10 on macrophages) work similarly as canonical “do not eat me” receptors (e.g., signal-regulatory protein alpha) (8). The functional parallels between Siglecs and immunomodulatory receptors, particularly regarding cancer immunity, have led to a recent surge in the interest in Siglecs and their ligands.

As the name implies, Siglecs recognize glycans containing sialic acid. Past extensive investigations have contributed to the establishment of Siglec glycan recognition specificities (1, 2, 4, 5). Some questions remain, however, such as whether and how these glycan epitopes (glycotopes) are displayed on natural glycoconjugates (glycoproteins and/or glycolipids) and which ligand is most significant in a given biological context. Affinity purification is often used to identify the ligand for a lectin. However, the inherently weak interaction between Siglec and the glycotope (dissociation constant usually in the order of 10^−3^ mol/L) renders affinity purification ineffective. Recent *in vitro* studies using innovative methodologies in chemical biology and/or genetics are beginning to reveal the Siglec ligand constituents in the cellular context.

**Figure 1:**
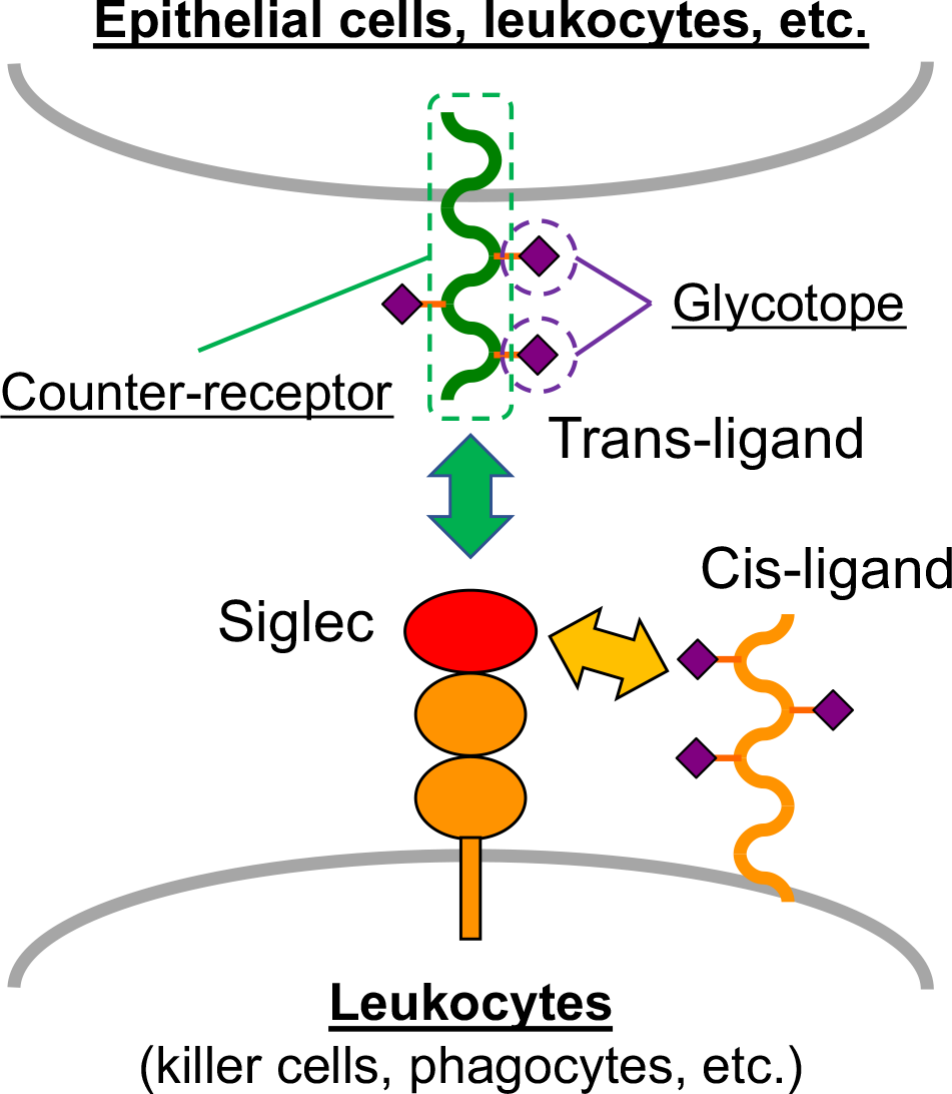
Siglec and Siglec ligand. Most Siglecs are expressed on leukocytes. The ligands on the same cell that express Siglec are called cis-ligands, and those on the juxtaposing cells (e.g., epithelial cells and other leukocytes) are called trans-ligands. Glycoprotein ligands of Siglecs comprise two constituents: glycotope, which directly interacts with Siglec, and counter-receptor, which is the protein backbone that displays the glycotope.

Siglec ligands can be classified into two categories (**Figure 1**): Siglec ligands expressed on the same cells that express the Siglec of interest (cis-ligands), and those on juxtaposing cells interacting with the cells on which Siglec of interest is expressed (trans-ligands). If a Siglec ligand is a glycoprotein, it comprises a glycan epitope being recognized by the Siglec of interest (glycotope), and the protein backbone that displays glycotope (counter-receptor).

In this Mini Review, we summarize recent methodological progress in the identification of physiologically relevant Siglec ligands in cellular contexts. Additionally, we discuss the advantages and disadvantages of these new approaches. For a comprehensive review of Siglec ligands, readers are encouraged to refer to a recent review (9).

## Methodologies to Identify Siglec Ligands

### Affinity Purification With a New Design of Recombinant Siglec Protein

For affinity purification, one has to prepare a solid phase matrix on which recombinant Siglec is immobilized, and use it to enrich Siglec ligand from cell lysate or biological fluid. Traditionally, the protein fusion “tag” of choice in recombinant Siglecs has been the fragment crystallizable (Fc) region of human immunoglobulin G (IgG). This choice is because it facilitates the folding of recombinant protein (thus increasing the yield). Additionally, the recombinant protein containing IgG-Fc can be easily purified with protein A resin (10). Nevertheless, IgG-Fc fusion protein is a homo-dimer (bivalent), which may not be sufficient to compensate for the low binding affinity between Siglec and its ligand by multivalency. An alternative protein fusion tag that allows the formation of a higher oligomer, thus increasing the “avidity,” may be useful for affinity purification. A novel protein tag [homo-pentamerization domain of cartilage oligomeric matrix protein (COMP)] has been adopted for the production of recombinant Siglec-8 protein, facilitating the identification of Siglec-8 ligand in the human airway (11). Whether the pentamer (as formed by COMP oligomerization domain) is optimal or other oligomer(s) may perform better is unknown as of yet. Artificially designed helical bundle oligomer tags, forming homo-tetramer, homo-pentamer, or homo-hexamer (12), may be useful to researchers endeavoring to answer this question.

A caveat of the affinity purification approach is that it requires a large amount of recombinant Siglec protein (usually in multimilligrams) for the preparation of the affinity matrix. Also, the affinity purification of integral membrane proteins serving as Siglec ligand requires disruption of the cell membrane by detergent or chaotropic ion, which inevitably dissociates cell surface protein complexes. Many Siglec ligands recently identified *via* affinity purification are soluble proteins (11, 13–15), likely because membrane solubilization leads to loss of the cell surface protein complex, which may be a prerequisite for Siglec –ligand interaction.

### Proximity Labeling

To overcome some of the limitations of the traditional affinity purification method, several groups have developed methods to identify Siglec ligands in a cellular context. One approach was to install a photoreactive sialic acid analog on cell surface glycoconjugates, followed by cross-linking and immuno-precipitation of the Siglec of interest. This process is followed by mass spectrometry-based proteomics to identify proteins that are cross-linked with the Siglec (16, 17). This approach revealed biologically relevant ligands for CD22/Siglec-2: CD22 itself as a major cis-ligand, and surface IgM as a major trans-ligand, of CD22. However, to apply this method to other Siglecs, one would have to evaluate whether the reactive group installed on sialic acid is tolerated by the Siglec of interest. Prior knowledge of the sialyltransferase responsible for the biosynthesis of the glycotope recognized by the Siglec may also be required. Hence, a more facile and versatile method may be needed.

Recently, some groups (including ours) developed methods to identify Siglec ligands on the basis of the same chemical principle: proximity labeling of proteins with short-lived tyramide radicals generated by peroxidase (18–20). This chemical principle has been known for decades and adapted for the enhancement of antibody-binding signals in immunohistochemical staining [known as catalyzed reporter deposition or tyramide signal amplification (21)]. However, its application for the identification of protein interacting partners (ligands and cluster) was only recently realized (22).

**Figure 2:**
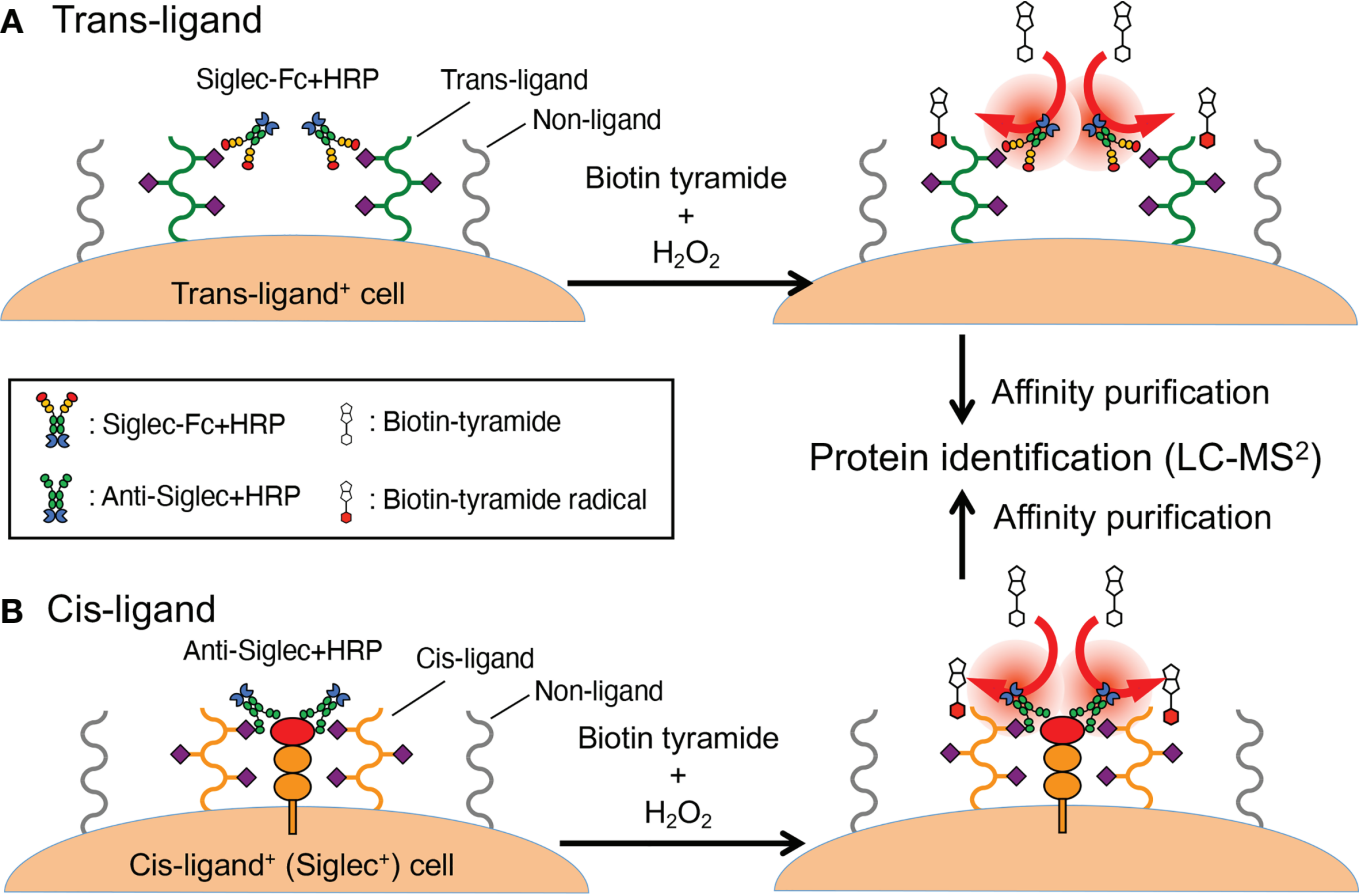
Proximity labeling method. **(A)** A workflow to identify trans-ligands. Cells that express Siglec ligand (as revealed by flow cytometry, microscopy, etc.) are labeled with a recombinant Siglec of interest that is coupled to peroxidase (either as a fusion protein or by way of complexing with a secondary reagent). The cells are washed and then exposed to biotin tyramide and hydrogen peroxide, which generates short-lived tyramide radicals that diffuse a limited distance from the origin before reacting with tyrosine residues in the vicinity (or diminish). This limited diffusion distance ensures selective labeling of the proteins in the proximity of the Siglec ligand, to which the Siglec–peroxidase complex is attached. **(B)** A workflow to identify cis-ligands. The probe used in this workflow is not recombinant Siglec but a peroxidase-coupled antibody that recognizes the Siglec of interest. Otherwise, the overall workflow is similar to **(A)**. In fact, the workflow described in **(A)** can also be applied for the identification of cis-ligand.

Here, cells expressing the Siglec ligand are incubated with peroxidase-coupled recombinant Siglec, followed by the addition of tyramide-based labeling compound (often biotin tyramide) and hydrogen peroxide. The addition of hydrogen peroxide generates short-lived tyramide radicals in the vicinity of the Siglec–peroxidase probe (thus in the vicinity of Siglec ligands) (**Figure 2A**). Coupling of peroxidase [horseradish peroxidase (HRP)] with Siglec can be achieved in one of two ways: by preparing Siglec–peroxidase fusion protein (19) or by combining Siglec–Fc with peroxidase-conjugated secondary antibody (18). Biotin-labeled proteins are purified by affinity purification from the cell lysate and identified by mass spectrometry-based proteomics. Studies utilizing this methodology demonstrated glycophorin A acts as a Sialoadhesin/Siglec-1 counter-receptor on human erythrocytes (19), and CD44 acts as a counter-receptor for Siglec-15 on RAW264.7 mouse macrophage cell line (18).

A variation of this protocol, applicable to the identification of cis-ligands (**Figure 2B**), is to use HRP-conjugated (or coupled) antibody against the Siglec of interest (20). This study yielded insight on the mechanism wherein CD22/Siglec-2 regulates B cell signaling. These results complemented results from past studies (23–26) and the chemical biology-based approach mentioned above (16). This method may be considered an implementation of the “selective proteomic proximity labeling using tyramide” method (27) targeting Siglecs.

Yet another variation of this method is to use another peroxidase (APEX) fused with the lectin of interest (28, 29). APEX is an engineered peroxidase developed from cytosolic ascorbate peroxidase of leguminous plants. It folds well in the cytosol of mammalian cells (whereas HRP fails to do so) (30, 31). APEX–galectin-3 fusion protein was used for the identification of both extracellular and intracellular interaction partners of galectin-3 (28). This study confirmed known interaction partners as well as revealed new partners. In principle, Siglec–APEX fusion protein would also be useful for the identification of Siglec ligands.

An advantage of the proximity labeling-based ligand identification approach is that it requires a relatively small amount of recombinant Siglec (on the order of micrograms). Additionally, the number of cells required is small (on the order of 10^6^ cells), making it possible to attempt the identification of Siglec ligands not only on cell lines but also on primary cells.

Some caveats of this approach may be as follows: [1] glycolipids are not labeled by tyramide radicals and thus cannot be identified; [2] some proteins poor in tyrosine, which is the primary amino acid labeled by tyramide radical (22), may not be labeled efficiently and thus may not be identified; [3] bystander proteins in the vicinity of true Siglec ligand are also labeled and identified; [4] some glycoproteins inherently resistant to proteolysis (such as mucins and mucin-like glycoproteins) may not be identified easily *via* mass spectrometry.

### Genetically Modified Cell Array

Recent advances in genetic tools, particularly CRISPR/Cas9-based genetic manipulation tools for gene editing and silencing, have been applied to modify glycosylation-related genes. Dr. Henrik Clausen’s group has developed extensive libraries of cell lines with modified glycosyltransferase genes. These lines were initially modified with zinc finger nucleases (32), and more recently with CRISPR/Cas9 (33). A recent publication from this group revealed the details of glycotopes recognized by Siglecs and glycosyltransferases involved in their synthesis. They also described the importance of galactose sulfation for the generation of glycotope recognized by several Siglecs (34), which was independently confirmed by another group (35). For several Siglecs, mucin-like glycoproteins appear to be effective counter-receptors.

Although this approach is no doubt powerful, it is not without caveats, as follows: [1] it is labor-intensive to develop and maintain a comprehensive library of cells comprising several sublines in which a single gene (or combination of genes) is disrupted and/or overexpressed; [2] the cell line used as the platform for the library may not be the best model of the cell type of interest.

### Genome-Wide Knockout/Knockdown Screening

An extension of the “cell library” approach is to utilize Cas9 and a single guide RNA (sgRNA) library to prepare an *ad hoc* library of gene-disrupted cells in mixture. After library creation, cells showing reduced (or enhanced) Siglec binding are enriched by cell sorting. Finally, researchers seek to identify the genes targeted in the cells (i.e., sgRNA enriched in the cells) that lost (or gained) Siglec binding. A recent study demonstrated this approach is feasible for the identification of Siglec ligands (36). This research revealed that a primary Siglec-7 counter-receptor on the K562 human erythroleukemia cell line is CD43. It also revealed that the cluster of O-glycans on the N-terminus of CD43 is important for recognition by Siglec-7. CD43 was independently confirmed by another group using proximity labeling as the Siglec-7 counter-receptor (37).

An advantage of this approach is that one can reveal unsuspected pathway(s) that regulate the expression of Siglec ligands, providing novel insights into the mechanism regulating Siglec – ligand interactions as well as possibly revealing a novel point of intervention for therapeutic applications. A genome-wide knockout/knockdown screening can, in theory, identify all the factors that contribute to the expression of Siglec ligands. Genome-wide screening using primary cells or live animals [using transgenic mice expressing Cas9 protein (38)] is possible, although a large amount of sgRNA-coding lentivirus may be required (39). A weakness of this approach is that it may not reveal genes essential for Siglec ligand expression in the presence of redundancy (e.g., multiple counter-receptors, alternative biosynthetic pathways, etc.).

## Discussion

Although significant methodological progress toward the identification of Siglec ligands in a cellular context has been made in recent years, there is no single method that applies to all biological contexts in which Siglecs are involved. A combination of new and traditional methods (such as glycosylation inhibitors and glycosidases), along with supportive bioinformatics, may prove most efficient in identifying biologically relevant ligands for Siglecs.

Some studies utilizing genome-wide knockout screenings to identify the genes influencing cancer cell sensitivity to NK cells (40), cytotoxic T cells (41), and antibody-dependent cellular phagocytosis by macrophages (42) have revealed some of the genes involved in the sialic acid biosynthetic pathway. Whether Siglecs are involved in the observed phenomenon is not clear. Nevertheless, in-depth analysis of gene lists obtained in these studies may reveal some interesting pathways influencing cancer immunoevasion *via* engaging Siglecs on killer leukocytes and phagocytes.

Identification of Siglec ligands, particularly counter-receptors, could lead to novel therapy options. For example, an antibody that recognizes a counter-receptor carrying a specific glycotope expressed on cancer cells (an equivalent of checkpoint ligand) may complement an immunotherapy that targets the cognate Siglec. One major obstacle in this direction is that, there is no established method to generate an antibody that recognizes a glycotope displayed on a specific protein scaffold. Technological breakthroughs and a platform enabling the development of such antibodies are highly anticipated (43).

## Author Contributions

TA conceptualized the content of the review and wrote the first draft. H-SJ, S-CZ, CHL, and L-YC performed literature search and revised the manuscript. All authors contributed to the article and approved the submitted version.

## Funding

This work is supported by intramural grants from Academia Sinica, Taiwan (AS-TP-108-ML06, AS-KPQ-110-BioMed, AS-GC-110-MD04, and AS-GC-111-M03).

## Conflict of Interest

The authors declare that the research was conducted in the absence of any commercial or financial relationships that could be construed as a potential conflict of interest.

## Publisher’s Note

All claims expressed in this article are solely those of the authors and do not necessarily represent those of their affiliated organizations, or those of the publisher, the editors and the reviewers. Any product that may be evaluated in this article, or claim that may be made by its manufacturer, is not guaranteed or endorsed by the publisher.
